# Anti-inflammatory and antioxidant activity of essential amino acid α-ketoacid analogues against renal ischemia-reperfusion damage in Wistar rats

**DOI:** 10.7705/biomedica.4875

**Published:** 2020-06-30

**Authors:** Concepción Sánchez-Martínez, Liliana Torres-González, Gabriela Alarcón-Galván, Linda E. Muñoz-Espinosa, Homero Zapata-Chavira, Diana Patricia Moreno-Peña, Homero Náñez-Terreros, Edelmiro Pérez-Rodríguez, Lourdes Garza-Ocañas, Francisco Javier Guzmán-de la Garza, Paula Cordero-Pérez

**Affiliations:** 1 Centro Regional de Enfermedades Renales, Departamento de Medicina Interna, Hospital Universitario “Dr. José E. González”, Universidad Autónoma de Nuevo León, Monterrey, México Centro Regional de Enfermedades Renales, Departamento de Medicina Interna, Hospital Universitario “Dr. José E. González” Universidad Autónoma de Nuevo León Monterrey México; 2 Unidad de Hígado, Departamento de Medicina Interna, Hospital Universitario “Dr. José E. González”, Universidad Autónoma de Nuevo León, Monterrey, México Unidad de Hígado, Departamento de Medicina Interna, Hospital Universitario “Dr. José E. González” Universidad Autónoma de Nuevo León Monterrey México; 3 Departamento de Ciencias Básicas, Facultad de Medicina, Universidad de Monterrey, San Pedro Garza García, México Departamento de Ciencias Básicas, Facultad de Medicina Universidad de Monterrey San Pedro Garza García México; 4 Servicio de Trasplantes, Hospital Universitario “Dr. José E. González”, Universidad Autónoma de Nuevo León, Monterrey, México Servicio de Trasplantes, Hospital Universitario “Dr. José E. González” Universidad Autónoma de Nuevo León Monterrey México; 5 Departamento de Medicina Interna, Hospital Universitario “Dr. José E. González”, Universidad Autónoma de Nuevo León, Monterrey, México Departamento de Medicina Interna, Hospital Universitario “Dr. José E. González” Universidad Autónoma de Nuevo León Monterrey México; 6 Departamento de Farmacología y Toxicología, Facultad de Medicina, Universidad Autónoma de Nuevo León, Monterrey, México Departamento de Farmacología y Toxicología, Facultad de Medicina Universidad Autónoma de Nuevo León Monterrey México; 7 Departamento de Fisiología, Facultad de Medicina, Universidad Autónoma de Nuevo León, Monterrey, México Departamento de Fisiología, Facultad de Medicina Universidad Autónoma de Nuevo León Monterrey México; 8 Centro de Investigación Biomédica del Noreste, Instituto Mexicano del Seguro Social, Monterrey, México Escuela de Enfermeria del Instituto Mexicano del Seguro Social Centro de Investigación Biomédica del Noreste Instituto Mexicano del Seguro Social Monterrey Mexico

**Keywords:** Ischemia, reperfusion injury, renal insufficiency, chronic, inflammation, oxidative stress, models, theoretical, isquemia, daño por reperfusión, insuficiencia renal crónica, inflamación, estrés oxidativo, modelos teóricos

## Abstract

**Introduction:**

Essential amino acid α-keto acid analogs are used in the treatment of chronic kidney disease to delay the symptoms of uremia. However, it is unknown whether essential amino acid α-keto acid analogs affect the oxidative stress and the inflammation in acute renal injury such as those produced by ischemia-reperfusion.

**Objective:**

To evaluate the effect of essential amino acid α-keto acid analogs on renal ischemia-reperfusion injury in Wistar rats.

**Materials and methods:**

Rats were divided into 11 groups (n=6/group): Two groups received physiological saline with or without ischemia-reperfusion injury (45 min/24 h), six groups received essential amino acid α-keto acid analogs (400, 800, or 1,200 mg/kg/24 h/7d) with or without ischemia-reperfusion injury (essential amino acid α-keto acid analogs + ischemia-reperfusion), and two groups received allopurinol (50 mg/kg/24 h/7d) with or without ischemia-reperfusion injury. Biochemical markers included creatinine and blood urea nitrogen (BUN), proinflammatory cytokines (IL-1β, IL-6, and TNF-α), renal damage markers (cystatin C, KIM-1, and NGAL), and markers of oxidative stress such as malondialdehyde (MDA) and total antioxidant activity.

**Results:**

The essential amino acid α-keto acid analog- and allopurinol-treated groups had lower levels of creatinine, BUN, renal damage markers, proinflammatory cytokines, and MDA than their corresponding ischemia-reperfusion groups. These changes were related to the essential amino acid α-keto acid analogs dosage. Total antioxidant activity was lower in essential amino acid α-keto acid analog- and allopurinol-treated groups than in the corresponding ischemia-reperfusion groups.

**Conclusions:**

This is a new report on the nephroprotective effects of essential amino acid α-keto acid analogs against ischemia-reperfusion injury. Essential amino acid α-keto acid analogs decreased the levels of biochemical markers, kidney injury markers, proinflammatory cytokines, and MDA while minimizing total antioxidant consumption.

## Resumen

**Introducción.:**

Los α-cetoanálogos de aminoácidos esenciales se utilizan en el tratamiento de la enfermedad renal crónica para retrasar los síntomas de la uremia. Sin embargo, se desconoce si los α-cetoanálogos de aminoácidos esenciales afectan el estrés oxidativo y la inflamación en la lesión renal aguda tal como en la producida por la isquemia-reperfusión.

**Objetivo.:**

Evaluar el efecto de las α-cetoanálogos de aminoácidos esenciales sobre la lesión renal por isquemia-reperfusión en ratas Wistar.

**Materiales y métodos.:**

Se emplearon 11 grupos de ratas (n=6): dos grupos recibieron solución salina fisiológica con lesión isquemia-reperfusión o sin ella (45 min/24 h), seis grupos recibieron α-cetoanálogos de aminoácidos esenciales (400, 800 o 1.200 mg/kg/24 h/7d) con lesión isquemia-reperfusión o sin ella (α-cetoanálogos de aminoácidos esenciales + isquemia-reperfusión), y dos grupos recibieron (50 mg/kg/24 h/7d) con lesión isquemia- reperfusión o sin ella. Los marcadores bioquímicos incluyeron creatinina y nitrógeno ureico en sangre (BUN), citocinas proinflamatorias (IL-1β, IL-6 y TNF-α), marcadores de daño renal (cistatina C, KIM-1 y NGAL) y marcadores del estrés oxidativo como el malondialdehído (MDA) y la actividad antioxidante total.

**Resultados.:**

Los grupos tratados con α-cetoanálogos de aminoácidos esenciales y alopurinol tuvieron niveles inferiores de creatinina, BUN, marcadores de daño renal, citocinas proinflamatorias, actividad antioxidante total y MDA que los grupos isquemia- reperfusión correspondientes. Estos cambios se asociaron con la dosis. La actividad antioxidante total fue menor en los grupos tratados con α-cetoanálogos de aminoácidos esenciales que en los grupos isquemia-reperfusión correspondientes.

**Conclusiones.:**

Este es un nuevo informe de los efectos nefroprotectores de las α-cetoanálogos de aminoácidos esenciales contra la lesión isquemia-reperfusión. Los α-cetoanálogos de aminoácidos esenciales disminuyeron los niveles de los marcadores bioquímicos, de los de lesión renal, de las citocinas proinflamatorias y el MDA, a la vez que minimizaron el consumo total de antioxidantes.

Essential amino acid α -keto acid analogs are used as conservative treatment of chronic kidney disease to reduce the production of metabolites of nitrogen degradation, improve the glomerular filtration rate, and delay the need for substitution treatment ^1^. The beneficial effect of essential amino acid α-keto acid analogs along with a low-protein diet in treating chronic kidney disease was first described in the 1970s ^2^ and the treatment is still used. A recent meta-analysis reported that essential amino acid α-keto acid analogs can delay the progression of chronic kidney disease by limiting hyperphosphatemia, preventing hyperparathyroidism, and improving the control of arterial blood pressure and malnutrition. Essential amino acid α-keto acid analogs are recommended in the treatment of stages 3 to 5 chronic kidney disease ^3^.

However, it is not clear whether essential amino acid α-keto acid analogs have other beneficial effects, especially in the early stages of chronic kidney disease or in acute kidney injury. Acute kidney injury can be caused by acute tubular necrosis as a result of various types of ischemia. Renal ischemia is observed in a variety of clinical situations, such as recovery after cardiac arrest, liver and kidney transplantation, and partial nephrectomy. Acute kidney injury observed after ischemia is characterized by decreased glomerular filtration rate, tubular necrosis, and increased renal vascular resistance. These changes can cause damage to tubular cells through the release of reactive oxygen species (ROS) and nitric oxide and an increased intracellular calcium concentration, which can in turn trigger mitochondrial damage through the depletion of antioxidants, cell-mediated cytotoxicity, and inflammatory response ^4^. These processes are similar to those occurring in chronic kidney disease, in which a series of events triggers the process of fibrosis or scarring through a cytokine-mediated inflammatory response that can activate extracellular matrix-producing cells in the glomerulus and tubules and lead to the repair or scarring of various renal components ^5^.

Ischemia-reperfusion injury occurs when the blood supply is cut off for a prolonged period and is then suddenly perfused with oxygenated blood ^6^. This process is characterized by the release of ROS and an intense inflammatory response provoked by the cellular response to the damaged tissue. Ischemia-reperfusion injury can be studied in animal models involving bilateral ischemia or heminephrectomy followed by ischemia of the remaining kidney. Bilateral ischemia is the preferred model because of its similarity to the pathological process in humans ^7^^,^^8^. Heminephrectomy has been used to evaluate the nephroprotective activity of drugs such as allopurinol, rosuvastatin, aliskiren, and rutin that are considered as nephroprotective ^8^^-^^11^.

Essential amino acid α-keto acid analogs may provide new agents for nephroprotection strategies, mainly for treating acute kidney injury and the early stages of chronic kidney disease. Understanding whether essential amino acid α-keto acid analogs have nephroprotective activity is the first step in developing new therapeutic strategies for the use of essential amino acid α-keto acid analogs as more than a nutritional supplement.

The principal objective of this study was to evaluate the effects of essential amino acid α-keto acid analogs on the inflammatory response and oxidative stress in a model of renal damage caused by ischemia-reperfusion injury in Wistar rats. As secondary objectives, we initially established the dose to be used not to generate hepatotoxicity or nephrotoxicity through biochemical markers and renal damage based on this established dose while the nephroprotective effect was evaluated through biochemical markers, proinflammatory mediators, and oxidative stress markers. Finally, we evaluated the effect of essential amino acid α-keto acid analogs on various typical markers of renal damage and histological changes in renal architecture post ischemia-reperfusion with and without administration of the selected dose of essential amino acid α-keto acid analogs.

## Materials and methods

### Animals

Experiments were performed using Wistar rats weighing 250-300 g in the *Departmento de Farmacología y Toxicología* of the *Facultad de Medicina* at *Universidad Autónoma de Nuevo León*, México. All animals were maintained in polypropylene cages at standard temperature (23-27°C) with 12 h light/dark periods and *ad libitum* access to water and standard food for rodents (Prolab diet 2500). Every experiment complied with the Mexican Official Norm NOM- 062-ZOO-1999 specifications and was approved by the committee for the institutional care and use of laboratory animals with the registration number (HI17-00001).

### Experimental design

**Figure 1:**
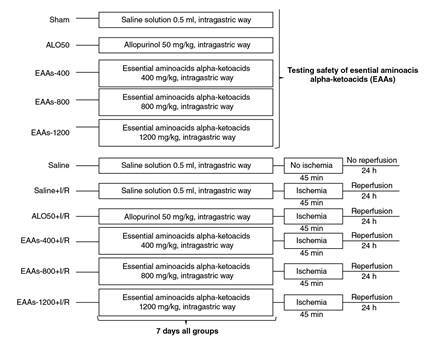
Experimental design of the study groups in the timeline

The animals were randomly divided into 11 groups with six rats in each group. The first five groups were used for evaluation of toxicity and groups 6 to 11 to evaluate nephroprotection. All doses evaluated were adjusted to a volume of 0.5 mL (figure 1).

The chemicals used were saline solution (CS PISA solution^™^, GDL México, sodium chloride 0.9%), allopurinol (Zyloprim^™^ tablets, Aspen Port Elizabeth Pty, Ltd, Reg. No. 86270 SSA IV), essential amino acid α-keto acid analogs (Cetolán Laboratorios Columbia^™^ Reg. No. 122M2016 SSA IV), xylazine (Sedaject, Vedilab S.A. de C.V. Reg. SAGARPA Q-0088-122), ketamine (Anesket, PiSA Agropecuaria, S.A. de C.V. Reg. SAGARPA Q7833-028).

The rats were anesthetized with xylazine (10 mg/kg, intraperitoneally (ip) and ketamine (100 mg/kg, ip). A small incision was made in the midline of the abdomen through which the renal pedicle was occluded with microvascular clamps; adequate occlusion was verified by cyanotic changes in both kidneys. The microvascular clamps were removed after 45 min to induce reperfusion, which was confirmed by verifying blood flow restoration. The abdominal incision was closed with a 4-0 suture. All experiments were performed under aseptic conditions.

The animals were monitored for 24 h and were allowed *ad libitum* access to water and food. The rats of the sham group received the same surgical procedure but without ischemia-reperfusion. Every animal was euthanized after obtaining blood and kidney tissue samples for evaluation.

### Blood and tissue samples

Blood was collected from each animal, allowed to clot, and centrifuged at 3,500 rpm for 15 min, and the serum was separated for later measurement of the concentrations of proinflammatory cytokines, biochemical markers, and kidney injury biomarkers. The kidneys were removed and divided longitudinally. One side was placed in a cryotube and immediately frozen in dry ice for later measurement of the concentrations of oxidative stress markers. The other side was fixed with formaldehyde in phosphate buffer (pH 7.4) for 24 h, embedded in paraffin, sectioned, and stained with hematoxylin and eosin to evaluate the histological alterations.

### Measurement of proinflammatory cytokine concentrations

Serum concentrations of the proinflammatory cytokines interleukin 6 (IL-6), interleukin 1-beta (IL-1β), and tumor necrosis factor-alpha (TNF-α) were measured using commercial sandwich ELISA kits (Rat IL-6 ELISA Development Kit^™^, Rat IL-1β ELISA Development Kit^™^, and Rat TNF-α ELISA Development Kit^™^, respectively, PeproTech, México City, México). Avidin-peroxidase and 2,2-azino-bis-3-ethylbenzthiazoline-6-sulfonic acid were used as the chromogen and the absorbance was measured by spectrophotometry at 405 nm.

### Biochemical markers

The serum concentrations of total proteins, albumin, urea nitrogen (BUN), creatinine, uric acid, aspartate aminotransferase (AST), alanine aminotransferase (ALT), lactate dehydrogenase (LDH), and alkaline phosphatase (ALP) were measured using an ILAB-Aries self-analyzer spectrophotometer and diagnostic kits (Instrumentation Laboratory, Milan, Italy) according to the supplier’s specifications.

### Kidney injury biomarkers

The serum levels of cystatin C (CysC), neutrophil gelatinase-associated lipocalin (NGAL), and kidney injury molecule-1 (KIM-1) were measured using commercial ELISA-sandwich kits (Quantikine ELISA, Mouse/Rat Cystatin C Immunoassay, R&D Systems, (Minneapolis, MN, USA), Lipocalin-2 (NGAL) Rat ELISA Kit, Abcam 119602, Cambridge, MA, USA, KIM-1 (TIM-1) Rat ELISA Kit, Abcam 119597, respectively) and then analyzed by spectrophotometry at 450 nm.

### Oxidative stress markers

Malondialdehyde (MDA) concentration was measured using a thiobarbituric acid-reactive substance (TBARS) assay and a TBARS Assay Kit^™^ (Cayman Chemical Co., Ann Arbor, MI, USA). The reaction product, which is proportional to the concentration of MDA in the sample, was measured spectrophotometrically at 540 nm. The total antioxidant activity was measured in homogenized kidney tissue using a commercial kit (Antioxidant Assay Kit^™^, Cayman Chemical Co.). Total antioxidant activity included both enzymatic and nonenzymatic activity and was measured by spectrophotometry at 405 nm.

### Statistical analysis

The results were expressed as mean ± standard deviation (SD). The homogeneity of variance was established and variables with a normal distribution were then analyzed using analysis of variance with multiple group comparisons through Dunnett’s test in the Prism software (v. 7.0; GraphPad, San Diego, CA, USA). A p-value of <0.05 was considered to be significant.

## Results

### Evaluation of toxicity

**Figure 2:**
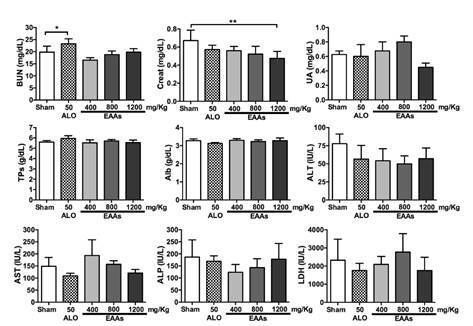
Changes in the serological levels of biochemical markers in toxicity study depending of treated groups (2-5) and sham control (Group 1). BUN: Blood urea nitrogen; Creat: Creatinine; UA: Uric acid; TPs: Total proteins; Alb: Albumin; ALT: Alanine aminotransferase; AST: Aspartate aminotransferase; ALP: Alkaline phosphatase; LDH: Lactate dehydrogenase; I/R: Ischemia-reperfusion; ALO: Allopurinol; EAAs: Essential amino acid α-ketoacid analogues Values are expressed as mean ± SD. * p<0.05, ** p<0.01

Essential amino acid α-keto acid analogs did not cause liver or renal toxicity when given at the different doses. The only markers that differed significantly between some of the study groups and the sham group were BUN, creatinine, NGAL, IL-1, and MDA levels (table 1, figure 2).

**Table 1:** Changes in the serological levels of biochemical markers of kidney damage and proinflammatory cytokines in the toxicity evaluation groups

Variable	Sham	ALO 50 mg/kg	EAA 400 mg/kg	EAA 800 mg/kg	EAA 1,200 mg/kg
CysC (ng/ml)	381.30±37.85	350.80±24.38	287.00±19.07	346.80±23.04	395.50±26.54
KIM-1 (pg/ml)	24.96±9.85	38.98±2.91	26.28±6.40	53.53±21.07	29.23±8.06
NGAL (pg/ml)	113.3±15.4	169.5±13.1*	159.0±11.9	104.0±5.5	170.0±14.1*
IL-1β (ng/ml)	0.35±0.19	0.41±0.09	0.82±0.06*	0.46±0.06	0.93±0.13*
IL-6 (ng/ml)	1.25±0.25	1.36±0.31	1.33±0.15	1.92±0.38	0.92±0.29
FNTα (ng/ml)	1.05±0.28	0.79±0.14	0.93±0.25	1.14±0.23	0.82±0.20
MDA (uM)	0.75±0.30	3.50±0.34***	1.46±0.25	1.96±0.39*	1.97±0.27*
AOxT (mM)	2.59±0.10	2.63±0.13	2.78±0.06	2.72±0.08	2.58±0.11

ALO: Allopurinol; EAA: Essential amino acid α ketoacid analogues; CysC: Cystatin C; KIM-1: Kidney injury molecule-1; NGAL: Neutrophil gelatinase-associated lipocalin; IL-1β: interleukin 1 beta; IL-6: interleukin 6; FNT-α: tumor necrosis factor alpha; MDA: malondialdehyde; AOxT: activity of the total antioxidants

Values are expressed as mean ± SD, sham group vs. study groups.

* p<0.05, ** p<0,01, *** p<0.001

### *Effects of essential amino acid* α-k*eto acid analogs on damage induced by ischemia-reperfusion injury*

**Figure 3.**
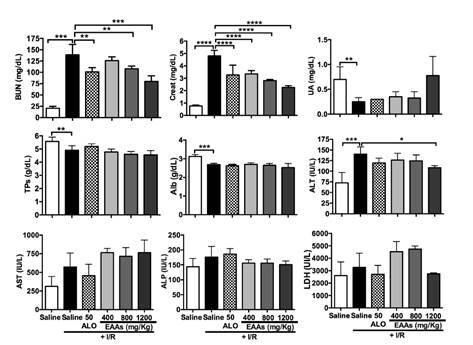
Changes in serum levels of biomarkers in the study of nephroprotection. BUN: Blood urea nitrogen; Creat: Creatinine; UA: Uric acid; TPs: Total proteins; Alb: Albumin; ALT: Alanine aminotransferase; AST: Aspartate aminotransferase; ALP: Alkaline phosphatase; LDH: Lactate dehydrogenase.; I/R: Ischemia-reperfusion; ALO: Allopurinol; EAAs: Essential amino acid α-ketoacid analogs Saline group + I/R (7) vs. sham group (6) and treated groups (8-11) Values are expressed as mean ± SD. * p<0.05, ** p<0.01, *** p<0.001, **** p<0.0001

*Biochemical markers.* Compared with the levels in the saline group, the levels of some biomarkers were significantly elevated in the saline + ischemia- reperfusion group: creatinine, BUN, and ALT (figure 3). The levels of other biomarkers were significantly lower in the saline + ischemia-reperfusion group than in the saline group: total proteins, albumin, and uric acid compared with the saline + ischemia-reperfusion group, groups allopurinol 50 + ischemia-reperfusion, essential amino acid α-keto acid analogs-800 + ischemia-reperfusion, and essential amino acid α-keto acid analogs-1,200 + ischemia-reperfusion had significantly lower creatinine and BUN levels. Essential amino acid α-keto acid analogs-400 + ischemia-reperfusion group also had lower creatinine and BUN levels, but the difference was significant only for creatinine level. Of the other biochemical parameters, only ALT concentration was significantly lower in the essential amino acid α-keto acid analogs-1200 + ischemia-reperfusion group than in the saline + ischemia-reperfusion group (figure 3).

### Kidney injury biomarkers

**Figure 4:**
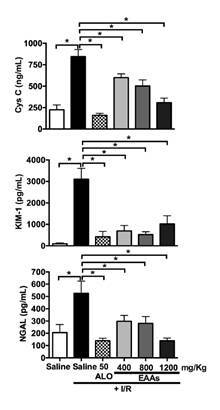
Changes in kidney injury biomarkers in the nephroprotection study. CysC: Cystatin C; KIM-1: Kidney injury molecule-1; NGAL: Neutrophil gelatinase-associated lipocalin.; I/R: Ischemia-reperfusion; ALO: Allopurinol; EAAs: Essential amino acid α-ketoacid analogs Saline group + I/R (7) vs. sham group (6) and treated groups (8-11) Values are expressed as mean ± SD. * p<0.0001

The levels of renal damage indicators (CysC, KIM-1, and NGAL) were significantly higher in the saline + ischemia-reperfusion group than in the saline group. These levels were significantly lower in the groups treated with allopurinol 50 and essential amino acid α-keto acid analogs at various doses than in the saline + ischemia-reperfusion group (figure 4).

### Proinflammatory cytokines

**Figure 5:**
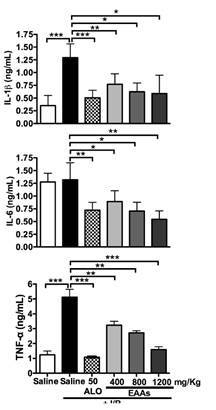
Changes in proinflammatory cytokines in the nephroprotection study. IL-1 β: Interleukin 1 beta; IL-6: Interleukin 6; TNF-α: Tumor necrosis factor alpha; I/R: Ischemia-reperfusion; ALO: Allopurinol; EAAs: Essential amino acid α-ketoacid analogues Saline group + I/R (7) vs. sham group (6) and treated groups (8-11) Values are expressed as mean ± SD. * p<0.01, ** p<0.001, *** p<0.0001

Compared with the saline group, the saline + ischemia-reperfusion group has significantly higher serum levels of the proinflammatory cytokines IL-1β and TNF-α. The control group showed a tendency to increase IL-6 levels compared to the sham group. Cytokine levels were significantly lower in the ALO50 + ischemia-reperfusion group than in the saline + ischemia-reperfusion group. A similar pattern was seen in groups treated with essential amino acid α-keto acid analogs at various doses (figure 5).

### Oxidative stress markers

**Figure 6:**
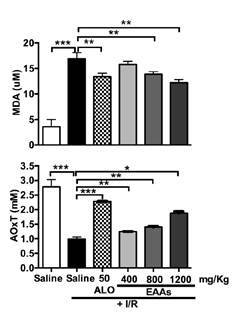
Changes in oxidative stress markers in the nephroprotection study. MDA: Malondialdehyde; AOxT: Activity of the total antioxidants; I/R: Ischemia-reperfusion; ALO: Allopurinol; EAAs: Essential amino acid α-ketoacid analogs Saline group + I/R (7) vs. sham group (6) and treated groups (8-11) Values are expressed as mean ± SD. * p<0.05, ** p<0.001, *** p<0.0001

Total antioxidant activity was significantly higher in the saline + ischemia- reperfusion group than in the saline group. Total antioxidant activity was significantly lower in the allopurinol 50 + ischemia-reperfusion and essential amino acid α-keto acid analogs + ischemia-reperfusion groups than in the saline + ischemia-reperfusion group, and this effect increased with the dose of essential amino acid α-keto acid analogs. The MDA level was significantly higher in the saline + ischemia-reperfusion group than in the saline-only group. The increment in MDA level was significantly smaller in the ALO50 + ischemia-reperfusion and essential amino acid α-keto acid analogs + ischemia-reperfusion groups than in the saline + ischemia-reperfusion group, and this effect increased with the dose of essential amino acid α-keto acid analogs (figure 6).

### Histological analysis

**Figure 7:**
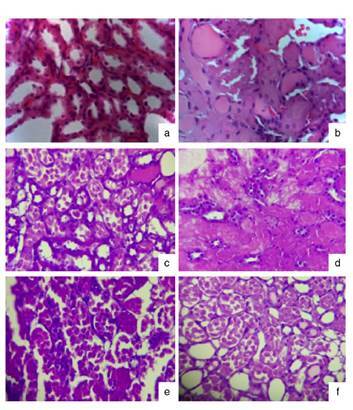
Histological findings in the study groups with and without damage by ischemia- reperfusion (H & E, 40X). **a)** S.S.: Preserved tubules. **b)** I/R: Acute tubular necrosis and intratubular cylinders. **c)** ALO + I/R: Medulla with acute tubular necrosis of convoluted tubules. **d)** EAAs 400+ I/R: Diffuse ischemic necrosis. **e)** EAAs 800+ I/R: Acute necrosis of juxtamedullary convoluted tubule. **f)** EAAs 1200 + I/R: Acute tubular necrosis of proximal tubules in medulla, preserved cortex I/R: Ischemia-reperfusion injury; ALO: Allopurinol; EAA: Essential amino acid α-ketoacid analogs

The saline-treated group showed normal renal parenchyma and preserved tubules and glomeruli, whereas the ischemia-reperfusion group showed necrosis of the tubular epithelium in 90% of the medulla and 80% of the cortex. The allopurinol 50 + ischemia-reperfusion group showed ischemic necrosis in 50% of the cortex and acute necrosis of the distal convoluted tubule. The other essential amino acid α-keto acid analogs + ischemia- reperfusion groups showed similar effects as those observed in the allopurinol 50 + ischemia-reperfusion group (figure 7).

### Discussion

Chronic kidney disease is currently considered a threat to global health as the number of patients increases rapidly ^12^. Chronic kidney disease is characterized by the development of glomerulosclerosis and interstitial fibrosis and involves the interactions of various factors such as angiotensin II, growth factors, cytokines, and oxygen metabolites ^13^.

### The restriction of proteins in the diet is an important therapy for people with chronic kidney disease.

Keto acid effects have been evaluated in patients with chronic kidney disease on a protein-restricted diet ^14^ but not in acute kidney injury patients. One study compared the efficacy of a low-supplemented protein diet (LPD) with keto acids (LPD + KA) and an LPD alone in stopping the development of kidney injury associated with chronic kidney disease in a 5/6 nephrectomy model in Sprague Dawley rats ^15^. This animal model of chronic kidney disease is characterized by proteinuria, decreased renal function, glomerular sclerosis, and tubulointerstitial fibrosis. Protein restriction decreased the extent of these changes. The effect was greater in the LPD + KA group than in the LPD group, and the LPD + KA group had lower serum levels of BUN and creatinine compared with the LPD group ^15^.

In our study, animals treated with various doses of essential amino acid α-keto acid analogs also had lower BUN and creatinine levels and this effect was dose dependent besides presenting normal liver enzyme levels, which means that at these doses the essential amino acid α-keto acid analogs were neither nephrotoxic or hepatotoxic. However, the levels of albumin, total proteins, and uric acid did not decrease. These findings suggest that essential amino acid α-keto acid analogs may have protected the kidneys from progressive injury by correcting the protein malnutrition in the remnant kidney tissue.

On the other hand, in the renal ischemia-reperfusion model, the levels of mediators of kidney damage, such as MCP-1, KIM-1, and CysC, increased with reperfusion time ^16^. We found that the levels of some of these damage mediators, such as KIM-1, CysC, and NGAL, decreased considerably in the essential amino acid α-keto acid analogs-treated groups that received the ischemia-reperfusion injury. These data support the nephroprotective effect of essential amino acid α-keto acid analogs.

Chronic kidney disease can be exacerbated by oxidative stress, which promotes the production of reactive carbonyl compounds and lipoperoxides that lead to advanced glycosylation accumulation and lipoxidation. ROS react with nitric oxide to produce reactive cytotoxic nitrogen species that are capable of producing nitrate proteins that can damage other molecules ^17^. Oxidative stress plays an important role in the development and progression of sclerosis and fibrosis in the remnant tissue in models of chronic renal failure ^17^^,^^18^. A low- protein diet, with or without keto acids, has an antioxidant effect in humans with chronic kidney disease and animal models of chronic kidney disease ^18^^,^^19^.

Previous studies in animals have found that essential amino acid α-keto acid analogs combined with a low-protein diet prevent weight loss, normalize albumin levels, maintain nutritional status, and improve the protein malnutrition and injury caused by oxidative stress in the remnant kidney tissue ^18^. Additionally, in rats submitted to 5/6 nephrectomy, the application of advanced oxidation protein products, which are associated with deterioration of renal function, imposes greater oxidative stress during the fibrotic process ^15^. Recently, the nephroprotective effect of essential amino acid α-keto acid analogs in the early stages of diabetic nephropathy type 2 was described mediated by the inhibition of oxidative stress through the decrease of MDA and the increase of superoxide dismutase ^20^.

In our study, we induced kidney damage in Wistar rats by ischemia- reperfusion and found that essential amino acid α-keto acid analogs modified the levels of oxidative stress markers as shown by the attenuated damage in the renal cortex and decreased total antioxidant activity and production of MDA in essential amino acid α-keto acid analogs/ischemia-reperfusion rats compared with ischemia-reperfusion rats. Again, these effects were essential amino acid α-keto acid analogs dose-dependent.

The factors that initiate the cascade of cell damage and the inflammatory response after ischemia-reperfusion leading to kidney damage are not completely understood. Increases in protein concentration have been described, such as the high-mobility group box-1 protein, which is released by kidney cells (particularly vascular and tubular cells) into the venous circulation after renal ischemia-reperfusion damage. This protein induces a rapid release of cytokines (TNF-α, G-CSF, IFN-Υ, IL-10, IL-1β, IL-6) into the systemic circulation ^21^^,^^22^. One study has reported decreases in the levels of IL-1β, IL-6, and in TNF-α in patients receiving peritoneal dialysis and treated with keto acids ^18^. In a rat experimental model with 5/6 nephrectomy treated with keto acids, IL-1β and IL-6 levels increased but TNF-α and IL-18 levels did not change significantly ^15^. We also observed decreases in the levels of IL-1β, IL-6, in TNF-α and the kidney damage markers CysC, KIM, and NGAL in the essential amino acid α-keto acid analogs-treated groups; this effect was dependent on the dose of essential amino acid α-keto acid analogs. These findings suggest that essential amino acid α-keto acid analogs decreased the inflammatory response to ischemia-reperfusion injury.

The inflammatory response is extremely important in the development of kidney damage; ischemia-reperfusion models are relevant to demonstrate anti-inflammatory effects resulting in delayed kidney damage. This has been demonstrated in several studies, such as the one published by Mori da Cunha, where they demonstrated in their ischemia-reperfusion model a nephroprotective effect of the application of amniotic fluid stem cells with increased regulation of vascular endothelial growth factor, which is dose- dependent ^23^. Other studies have also demonstrated the relationship of other inflammatory markers in the use of essential amino acid α-keto acid analogs, for example those involved in mineral and bone metabolism disorders mediated by FGF-23 and Klotho involved in inflammation, oxidative stress, and energetic protein malnutrition in stages 3b and 4 of chronic renal disease associated with a low-protein diet.

The nephroprotective effect in acute damage observed in our study has not been previously reported. This study opens up the possibility of essential amino acid α-keto acid analogs efficacy in both acute and chronic kidney disease damage ^24^.

In conclusion, we show that essential amino acid α-keto acid analogs have a nephroprotective effect against renal ischemia-reperfusion damage. Essential amino acid α-keto acid analogs decreased the levels of biochemical markers, markers of kidney damage, proinflammatory cytokines, and MDA, an effect that was related to the dose of essential amino acid α-keto acid analogs. Essential amino acid α-keto acid analogs exerted these protective effects while avoiding the consumption of total antioxidants. There is the possibility of a continuous protective effect even without a restriction of proteins, which would strengthen its use in early stages of chronic kidney disease, as well as its potential application in acute kidney damage and transplantation of solid organs to reduce injury by ischemia-reperfusion or improve the response in non-optimal organs for which studies are required.
